# Calcium-Dependent Calcium Decay Explains STDP in a Dynamic Model of Hippocampal Synapses

**DOI:** 10.1371/journal.pone.0086248

**Published:** 2014-01-22

**Authors:** Dominic Standage, Thomas Trappenberg, Gunnar Blohm

**Affiliations:** 1 Department of Biomedical and Molecular Sciences and Center for Neuroscience Studies, Queen’s University, Kingston, Ontario, Canada; 2 Faculty of Computer Science, Dalhousie University, Halifax, Nova Scotia, Canada; Instituto de Neurociencias de Alicante UMH-CSIC, Spain

## Abstract

It is widely accepted that the direction and magnitude of synaptic plasticity depends on post-synaptic calcium flux, where high levels of calcium lead to long-term potentiation and moderate levels lead to long-term depression. At synapses onto neurons in region CA1 of the hippocampus (and many other synapses), NMDA receptors provide the relevant source of calcium. In this regard, post-synaptic calcium captures the coincidence of pre- and post-synaptic activity, due to the blockage of these receptors at low voltage. Previous studies show that under spike timing dependent plasticity (STDP) protocols, potentiation at CA1 synapses requires post-synaptic bursting and an inter-pairing frequency in the range of the hippocampal theta rhythm. We hypothesize that these requirements reflect the saturation of the mechanisms of calcium extrusion from the post-synaptic spine. We test this hypothesis with a minimal model of NMDA receptor-dependent plasticity, simulating slow extrusion with a calcium-dependent calcium time constant. In simulations of STDP experiments, the model accounts for latency-dependent depression with either post-synaptic bursting or theta-frequency pairing (or neither) and accounts for latency-dependent potentiation when both of these requirements are met. The model makes testable predictions for STDP experiments and our simple implementation is tractable at the network level, demonstrating associative learning in a biophysical network model with realistic synaptic dynamics.

## Introduction

Activity-dependent change in synaptic strength is widely believed to underlie learning and memory, as originally proposed by Hebb [1]. To have behavioural significance, synapses must be equipped to extract statistical regularities in the spiking behaviour of their pre- and post-synaptic neurons. Numerous algorithms have implemented this principle in artificial neural networks, where the strength of connections between processing units changes as a function of correlations in their output rates [2]–[6]. These algorithms readily learn to associate patterns of network activity with one another, that is, they implement Hebbian associative learning (see [7]). Similarly, activity-dependent change in the strength of synapses, or synaptic plasticity, is well established in the forms of long term potentiation (LTP) [8] and long term depression (LTD) [9]. Notably, the direction of plasticity (LTP or LTD) has been shown to depend on pre- and post-synaptic activation [10], [11], including pre- and post-synaptic spike timing correlations (see [12]).

Spike timing-dependent plasticity (STDP) refers to experimental data showing that the direction and magnitude of synaptic change can be controlled by the precise timing of repeated pre- and post-synaptic spike patterns [13]–[19]. STDP has played an important role in revealing the mechanisms underlying plasticity (see [20], [21]), but ‘learning rules’ fit to these data do not easily generalize to deviations from the strict protocols under which the data were recorded (see [22]). In contrast, by simulating the underlying neurophysiology, synaptic models have provided mechanistic explanations for data recorded under different STDP protocols and at different synapses [23]–[30]. The fundamental premise of these models is that a biological variable captures the statistics of pre- and post-synaptic spiking and is used by intracellular signalling pathways to modify synaptic strength accordingly.

Here, we focus on the synapse that has been most intensively studied in plasticity experiments to date, that between pyramidal neurons in regions CA3 and CA1 of the hippocampus [22]. As with virtually all synapses, plasticity at CA3-CA1 synapses depends on post-synaptic calcium (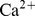) in dendritic spines, where high levels of 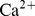 lead to LTP, and moderate, above-baseline levels lead to LTD (see [22], [31], [32]). We follow the approach of Shouval and colleagues [23] by further assuming that the relevant supply of 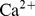 is mediated by NMDA receptors (NMDARs), known to be required for LTP and LTD at synapses onto CA1 neurons [32]. In this regard, NMDARs provide a mechanism for pre- and post-synaptic coincidence detection: they bind glutamate, providing a marker for pre-synaptic spiking, but they are blocked by magnesium until sufficiently depolarized by back-propagating action potentials (BAPs), which provide markers for post-synaptic spiking. 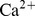 enters the post-synaptic spine when these two markers overlap.

Under STDP protocols, LTP at CA3-CA1 synapses not only depends on 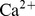 flux and NMDAR activation, but also requires post-synaptic bursting and an inter-pairing frequency in the range of the hippocampal theta rhythm [19] (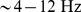). We build on earlier modelling work [23]–[30], [33] by proposing a novel mechanistic explanation for these two requirements for LTP. We hypothesize that these requirements reflect the saturation of the mechanisms underlying 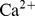 extrusion from the post-synaptic spine [34], [35], supporting the buildup of 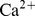 by preventing its decay with sufficiently-vigorous spiking activity. We test this hypothesis with a dynamic model of post-synaptic 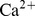 flux, where the rate of 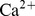 decay is 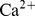-dependent. We show that our model accounts for the post-synaptic burst-dependence and theta-frequency pairing-dependence of LTP under spike timing protocols at CA3-CA1 synapses [19], [36]; we demonstrate that under biophysically plausible parameter values, the model cannot reproduce these findings without the dynamic 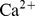 time constant; and we make predictions for experimental verification. We further demonstrate that under the same parameters that reproduce these data, our model supports Hebbian associative learning in a biophysical network model of hippocampal circuitry with realistic synaptic dynamics.

## Methods

Our model is based on the premise that high levels of post-synaptic 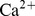 trigger kinase-activated intracellular pathways, leading to LTP, and moderate, above-baseline levels trigger phosphatase-activated pathways, leading to LTD [31], [37], [38]. Consistent with evidence that plasticity at CA1 synapses is NMDAR-dependent [32], we assume that NMDARs provide a coincidence detector of pre- and post-synaptic activity, permitting the influx of 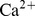 when their activation coincides with BAPs [23]. We take a simple approach in this regard, simulating the activation of NMDARs as the proportion of glutamate-bound channels at the synapse, simulating BAPs as the proportion of maximum dendritic depolarization, and simulating 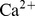 flux as the decaying product of these two terms, scaled by a soft cap. Note that our model addresses homosynaptic plasticity, implicit in the assumption of coincidence detection by NMDARs. Like earlier authors [23], [28], [30], [33], we assume that the tail of the BAP has a residual buildup, so that the overlap between NMDARs and BAPs supports 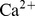 flux when post-synaptic spikes precede pre-synaptic spikes during STDP protocols (see below).

To test the hypothesis that the post-synaptic burst-dependence and inter-pairing frequency dependence of LTP at CA3-CA1 synapses can be explained by the saturation of the mechanisms of 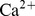 extrusion from the spine, we modelled the saturation of these mechanisms with a 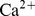-dependent 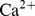 time constant. As such, the time constant of decay was modelled as the summation of a baseline time constant and a sigmoidal function of 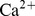. We directly simulated the STDP experiments of [19] by generating pre- and post-synaptic impulses according to their experimental protocols, as well as experiments from two earlier, related studies of CA3-CA1 synapses [15], [36].

To demonstrate that our 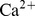-based plasticity model can support Hebbian associative learning under biologically plausible conditions, we embedded the model in a network of leaky integrate-and-fire neurons with realistic synaptic dynamics. Our chosen task was auto-associative recall, a classic task for models of hippocampal circuitry [39], [40].

### A 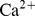-based Plasticity Model with a 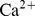-dependent 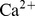 Time Constant

The activation of NMDARs is described by
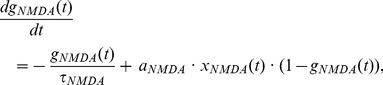
(1)where 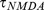 is the time constant of decay, 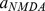 controls receptor saturation and 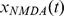 is the opening of receptor channels. Channel opening is described by
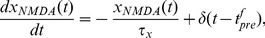
(2)where 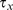 determines rise time, 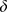 is the Dirac delta function and 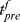 is the time of pre-synaptic firing.

BAPs are composed of a peak and a tail [23], [28], [30], [33]. The peak is defined by
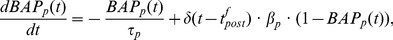
(3)where 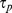 is the time constant of decay, 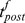 is the time of post-synaptic firing, and 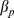 is a scale factor determining the proportion of the BAP magnitude attributable to the peak.

**Figure 1:**
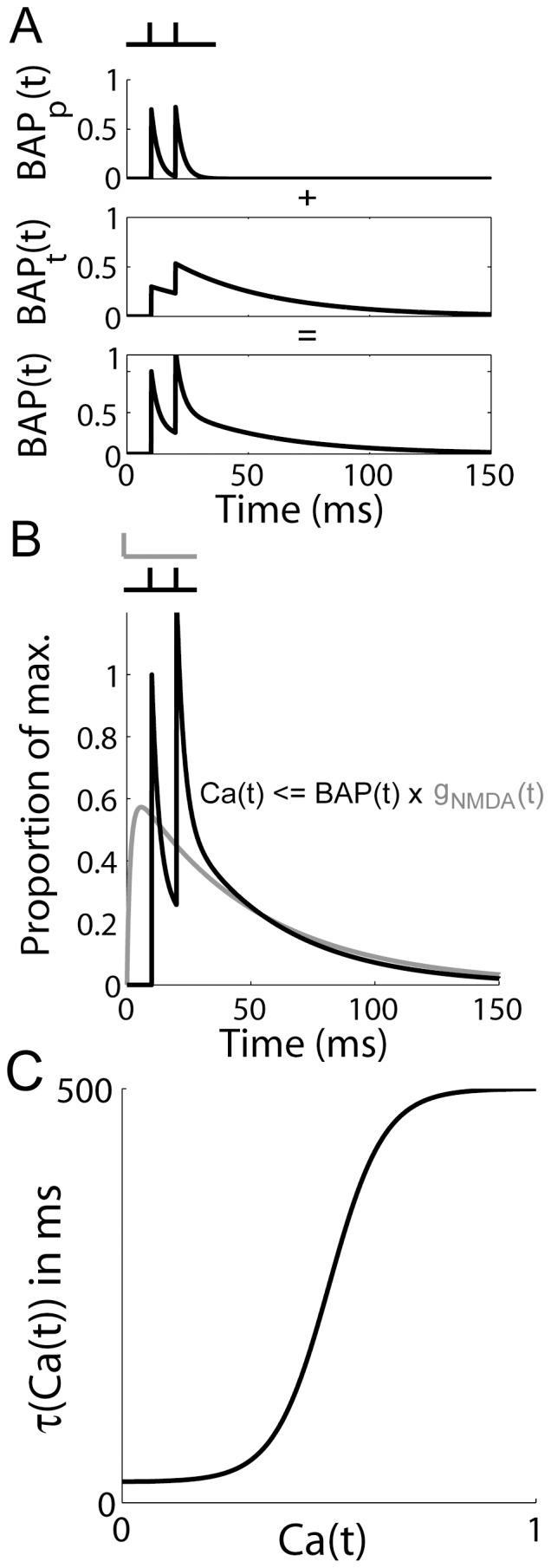
Minimal 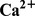 plasticity model with 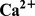-dependent 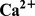 time constant. (A) Simulated back-propagating action potentials (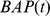, bottom) are composed of a peak (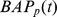, top) and a tail (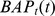, middle). The two vertical dashes above the figure indicate the timing of post-synaptic spikes. (B) The 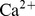-like variable 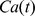 is the scaled product of NMDAR activation and the BAP (see Methods). Grey and black vertical dashes indicate the timing of pre- and post-synaptic spikes respectively. (C) 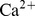-dependent 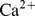 time constant 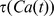, capturing the saturation of the mechanisms of 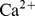 extrusion at high 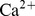 levels.

The BAP tail is defined by
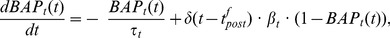
(4)with half-life 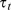 and scale factor 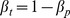. The composite BAP is defined by 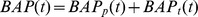, shown in Figure 1A. In simulations with more than one post-synaptic spike in each pairing, we allow the BAPs to summate, as seen during theta-burst stimulation of CA1 neurons [41].

The level of post-synaptic 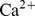 is simulated by
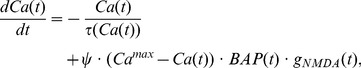
(5)where 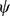 is a scale factor, Ca^*max*^=1 provides a cap on post-synaptic 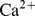 and 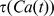 is a 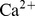-dependent 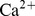 time constant, capturing the slow extrusion of high levels of 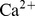 from the spine [35]. 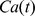 is depicted in Figure 1B. We use a sigmoid function for 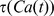, defined by
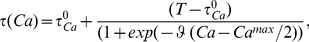
(6)where 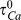 serves as a baseline time constant for post-synaptic 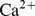 decay, 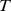 places an upper limit on 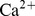 decay time, and 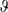 determines the slope of the function (Figure 1C).

Plasticity is determined by simultaneous LTP and LTD processes [18]. Our LTP rule is
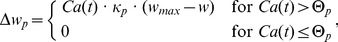
(7)where 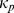 is the potentiation learning rate, 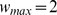 provides a maximum weight (conductance strength) for synaptic weight 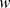 (given initial value 1), and 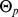 is the 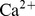 threshold for potentiation.

Our LTD rule is
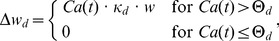
(8)where 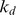 is the depression learning rate and 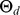 is the 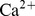 threshold for depression.

#### Parameter values

For NMDARs, our parameter values were 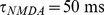 [42], [43], 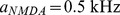 and 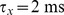 [44], [45]. For BAPs, we followed an earlier 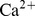-based plasticity model [33] by setting 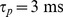, 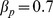 and 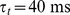, justified by data from CA1 slices [46]. For the potentiation and depression learning rates, we set 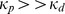, consistent with experimental data showing that LTD takes much longer to elicit than LTP [9], [19]. The chosen values were 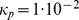 and 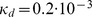. For the LTP threshold, we chose a high value to emphasize the robustness of the proposed mechanism, *i.e.* with 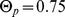, 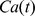 was far from the threshold when the inter-pairing frequency and post-synaptic burst requirements of LTP were not satisfied (shown in the Results). For the 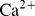-dependent 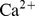 time constant 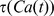, we chose a saturating function because our model captures a saturating process (extrusion of 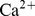 from the spine). The baseline 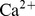 time constant was 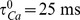 [34] and the maximum 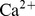 time constant was 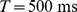, previously reported as the time constant of recovery from saturated extrusion in the spine at CA1 synapses [35]. We set the slope parameter 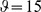 to a moderate value (see Figure 1C). The only remaining parameters were the LTD threshold 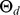 and the scale factor 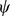, which we adjusted to produce a good approximation of the LTP data by [19] for 5 Hz pairings of a single pre-synaptic spike with two post-synaptic spikes (shown in the Results). The chosen values were 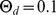 and 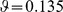, which we used in our simulations of all other protocols. Note that within a reasonable range (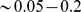), the LTD threshold controlled the width of the LTD window as a function of the latency between pre- and post-synaptic activity, but did not qualitatively effect our results.

It is worth noting that the baseline time constant of calcium decay (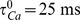) refers to 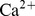 extrusion from the spine, which dominates 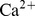 clearance at synapses onto pyramidal neurons (see [47]). Measurement of 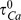 is achieved by visualizing calcium transients with 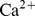-sensitive fluorescent molecules (calcium indicators), *i.e.* the indicators bind calcium so the transients can be measured. However, the binding of 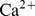 by indicators lengthens the duration of decay by providing an ‘exogenous buffer’ for 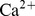, which slows its clearance. As such, numerous studies have reported 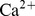 time constants on the order of hundreds of milliseconds, but these measurements included the effects of indicators. The time constant of 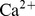 decay can be estimated by using multiple concentrations of indicator, measuring the time constant of decay for each concentration, and regressing to the case of 0-indicator. As such, the baseline time constant of 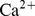 decay in dendritic spines is estimated to be several tens of milliseconds [34], [48]. At high concentrations of spine 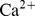, the mechanisms of extrusion saturate [35], providing the basis for our model. See [47] for a thorough review of these and related issues.

### The Network Model

The local circuit model is a fully recurrent network of leaky integrate-and-fire neurons [49], comprised of 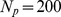 simulated pyramidal neurons and 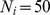 fast-spiking inhibitory interneurons. Excitatory currents from pyramidal neurons were mediated by AMPA receptor (AMPAR) and NMDAR conductances, and inhibitory currents from interneurons were mediated by GABA receptor (GABAR) conductances. Synaptic connections from pyramidal neurons to interneurons, from interneurons to pyramidal neurons, and from interneurons to interneurons were uniform. Synaptic connections from pyramidal neurons to pyramidal neurons were scaled by weight 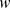, set to 0.5 at the start of each trial.

Each model neuron is described by
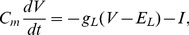
(9)where 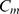 is the membrane capacitance of the neuron, 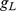 is the leakage conductance, 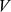 is the membrane potential, 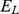 is the equilibrium potential, and 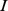 is the total input current. When 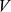 reaches a threshold 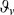, it is reset to 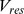, after which it is unresponsive to its input for an absolute refractory period of 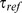. For pyramidal neurons, 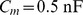, 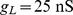, 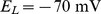, 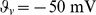, 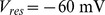 and 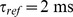. For interneurons, 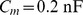, 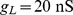, 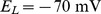, 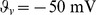, 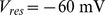 and 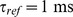 [44].

The total input current to each neuron 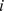 is given by
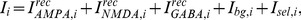
(10)where 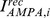, 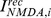 and 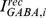 are the summed AMPAR, NMDAR and GABAR currents from intrinsic (recurrent collateral) synapses, 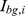 is background activity and 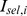 is selective input. The intrinsic currents are defined by
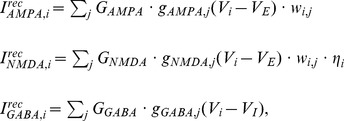
(11)where 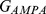, 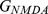 and 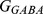 are the respective strengths of AMPAR, NMDAR and GABAR conductance, 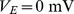 is the reversal potential for AMPARs and NMDARs, 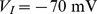 is the reversal potential for GABARs, and indeces 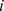 and 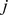 indicate the synaptic connection to pyramidal neuron 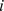 from pyramidal neuron 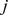. AMPAR and GABAR activation (proportion of open channels) are described by
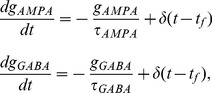
(12)where 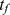 is the time of firing of a pre-synaptic neuron. NMDAR activation is described above in Equation 1. The voltage-dependence of NMDARs is captured by 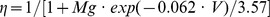, where 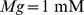 describes the extracellular magnesium concentration and 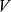 is measured in millivolts [50].

At intrinsic synapses onto pyramidal neurons, 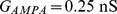, 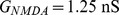 and 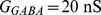. At synapses onto interneurons, these conductances were scaled by a factor of 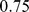, a common approach in this class of network [44], [45], [51], [52]. At extrinsic (feedforward) synapses onto pyramidal neurons, 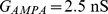, supporting strong selective input to the network. Consistent with recordings in hippocampal slices, the synaptic time constants used were 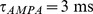 [53] and 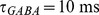 [54] (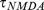 is provided above).

#### Background activity

Theta-rhythmic background activity was simulated by sinusoidal current injection. The current injected at each neuron was the product of a 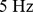 sin wave and the point conductance model of [55]. For each neuron, current 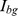 is defined by
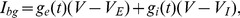
(13)where reversal potentials 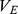 and 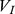 are given above (Equation 11). The time-dependent excitatory and inhibitory conductances 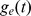 and 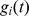 are updated at each timestep 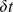 according to



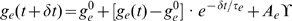
(14)and
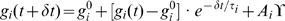
(15)where 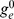 and 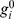 are average conductances, 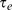 and 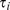 are time constants, 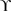 is normally distributed random noise with 0 mean and unit standard deviation. Amplitude coefficients 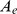 and 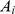 are defined by



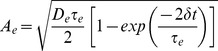
(16)and
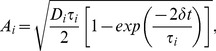
(17)where 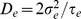 and 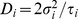 are noise ‘diffusion’ coefficients. See [55] for the derivation of these equations. We followed Table 1 of [55] for parameter values 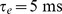, 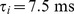, 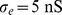 and 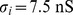 for pyramidal neurons and interneurons and 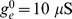 for pyramidal neurons. We gave nominal values to 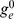 for interneurons and to 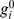 for pyramidal neurons and interneurons, setting these conductances to 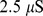, *i.e.* the network’s intrinsic connectivity was sufficient to mediate realistic levels of inhibitory background activity onto pyramidal neurons and excitatory and inhibitory background activity onto interneurons. All simulations were run with timestep 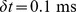 and the standard implementation of Euler’s forward method. Our results were verified with the standard fourth order Runge-Kutta method.

**Table 1:** A comparison of 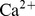-based plasticity models.

Model	NM	CT	IP	SS	CE	TD	LM
Shouval et al. (2002)	yes	int	no	yes	no	sym	no
Karmarkar and Buonomano (2002)							
Model 1	no	peak	no	yes	no	sym	no
Model 2	no	peak	no	no	no	asym	no
Abarbanelet al. (2003)	no	time	no	yes	no	sym	no
Rubin et al. (2005)	no	time	no	yes	no	both	no
Hartley et al. (2006)	yes	int	no	no	no	both	yes
Rackham et al. (2010)	yes	peak	no	yes	no	sym	no
Kumar and Mehta (2011)	yes	int	no	yes	no	sym	no
Graupner and Brunel (2012)							
Linear model	no	int	yes	yes	no	both	no
Nonlinearmodel	no	int	yes	yes	no	asym	no
Bush and Jin (2012)	yes	int	yes	yes	no	sym	no
Our model	yes	int	no	yes	yes	sym	yes

A comparison of 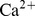-based plasticity models. In relation to our model, earlier models can be distinguished along dimensions including: whether NMDARs provide the only source of calcium for plasticity (NM: yes/no); whether plasticity outcomes are determined by integrated 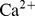, peak 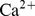 or the timecourse of 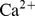 transients (CT: int/peak/time); whether an intermediate process transforms calcium levels to plasticity outcomes (IP: yes/no); whether LTP and LTD depend on the same source of 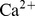 (SS: yes/no); whether saturation of the mechanisms of 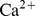 extrusion was simulated (CE: yes/no); whether the model accounts for symmetric and/or asymmetric STDP data (TD: sym/asym/both); and whether the model demonstrated learning and memory (LM: yes/no).

## Results

Before showing our results, it is useful to summarise the data being addressed. [19] recorded at CA3-CA1 synapses in slice preparations, where repetitive pairings of a single pre-synaptic spike with a single post-synaptic spike (the doublet protocol) produced LTD, the magnitude of which was inversely related to the time between spikes 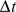. This was the case regardless of the order of pre- and post-synaptic spiking (the sign of 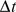) or the inter-pairing frequency (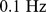, 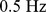 and 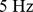). LTP not only required repetitive low-latency pairings of a pre-synaptic spike with at least two post-synaptic spikes (the triplet protocol), but also required an inter-pairing frequency of 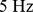. As with the doublet protocol, negative latencies produced LTD under the triplet protocol, the magnitude of which was inversely related to latency. Notably, these conditions also produced an LTD window at intermediate positive latencies, that is, LTD occurred when a post-synaptic burst followed a pre-synaptic spike by several tens of milliseconds. These data suggest temporally *symmetric* learning during the hippocampal theta rhythm, where tight coincidence of burst firing yields LTP, loose coincidence yields LTD, and a lack of coincidence yields no synaptic change (see [22]). Note that these constraints fit naturally with auto-associative models of hippocampal learning [39], [40], [56], demonstrated in Results section *Hebbian associative learning in the network model*. We refer to this form of STDP as temporally symmetric, bidirectional STDP. We refer to STDP data in which pre-before-post pairings lead only to LTP and post-before-pre pairings lead only to LTD as temporally *asymmetric*, bidirectional STDP. In summary, [19] ran experiments with four combinations of spike pairings and inter-pairing frequencies: (1) the doublet protocol at 0.1 Hz and 0.5 Hz produced LTD; (2) the doublet protocol at 5 Hz produced LTD; (3) the triplet protocol at 0.5 Hz produced LTD; and (4) the triplet protocol at 5 Hz produced temporally symmetric, bidirectional STDP. Thus, LTP required short-latency pairings in the range of theta and post-synaptic bursting. LTD was produced if either of these requirements was not met.

In addition to the data by [19], we also addressed data from two earlier plasticity studies with inter-pairing frequencies in the theta range, one demonstrating the requirement of post-synaptic bursting for LTP at CA1 synapses under protocols different than those described above [36], and one showing temporally symmetric, bidirectional STDP at CA1 synapses without the requirement of post-synaptic bursting [15]. In the latter case, we demonstrate that the physiological explanation provided by [19] for the lack of burst-dependence found by [15] accounts for these data in our model. All these results were produced under the same parameters.

### Temporally Symmetric STDP at CA3-CA1 Synapses

**Figure 2:**
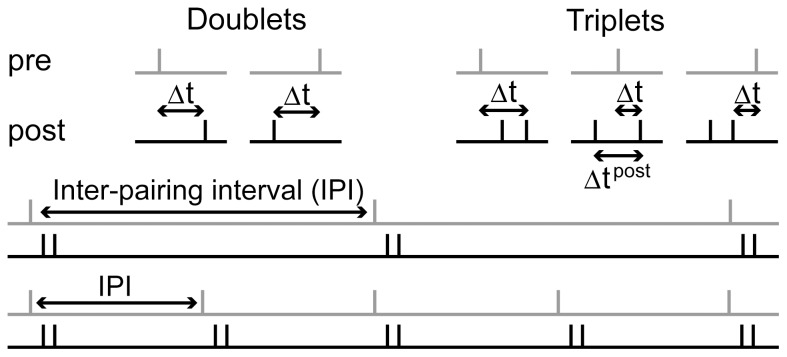
Spike timing dependent plasticity (STDP) protocols. STDP protocols used by [19] and corresponding terminology used here. For the doublet protocol, 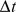 refers to the time between the pre-synaptic spike and the post-synaptic spike at each pairing. Pairings are referred to as pre-post (left) and post-pre (right). For the triplet protocol, 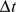 refers to the time between the pre-synaptic spike and the second post-synaptic spike. Pairings are referred to as pre-post-post (left), post-pre-post (middle) and post-post-pre (right). The time between post-synaptic spikes is referred as to 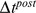. The inter-pairing frequency is the reciprocal of the time between pairings, the inter-pairing interval. Two spike trains are depicted for pre-post-post pairings, with low (upper) and high (lower) inter-pairing frequency respectively.

To determine whether our 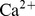 plasticity model can account for the data, we followed three STDP protocols used by [19]. First, we conducted simulations of the doublet protocol at latencies 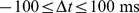, using inter-pairing frequencies of 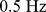 and 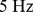. We refer to these pairings as pre-post for 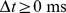 and post-pre for 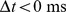. Next, we simulated the triplet protocol (a single pre-synaptic spike and two post-synaptic spikes) over the same range of latencies 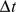, using an inter-pairing frequency of 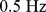. In this case, 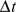 refers to the time between the pre-synaptic spike and the second post-synaptic spike. The post-synaptic spikes were separated by 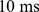. We refer to these pairings as pre-post-post for 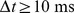, post-pre-post for 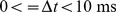, and post-post-pre for 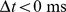. Finally, we repeated the triplet protocol with an inter-pairing frequency of 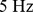. For all three sets of simulations, we used 75 pairings, consistent with the corresponding experiments ([19] used 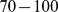 pairings in different conditions). These protocols and corresponding terminology are depicted in Figure 2.

**Figure 3:**
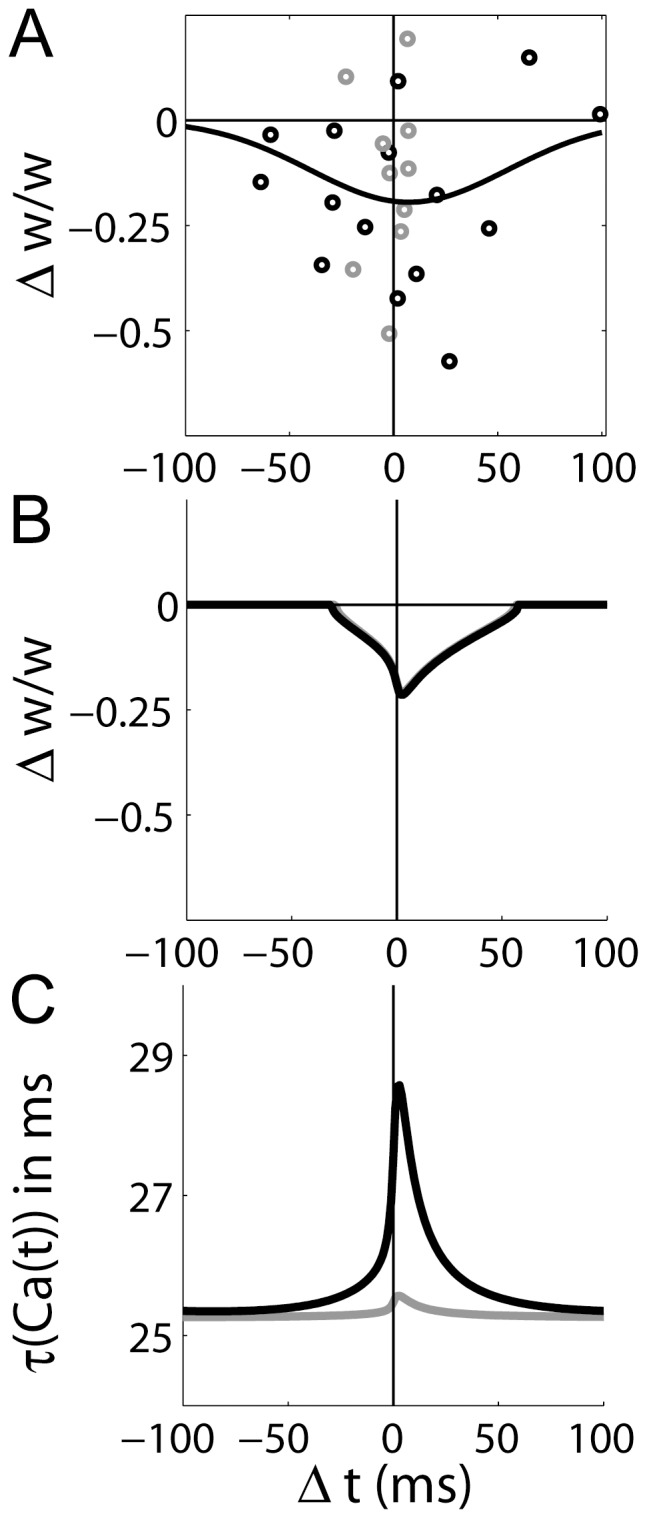
STDP at CA3-CA1 synapses with single pre- and post-synaptic spikes (the doublet protocol). (A) Data reproduced from [19], fit with a Gaussian function (least squares fit). The grey dots indicate an inter-pairing frequency of 0.1 Hz and 0.5 Hz. The black dots indicate an inter-pairing frequency of 5 Hz. 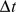 refers to the time between the pre-synaptic spike and the post-synaptic spike. 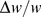 refers to the change in synaptic strength relative to initial strength. (B) The model produced latency-dependent LTD-only curves (0.5 Hz grey, 5 Hz black). The curves are nearly indistinguishable. (C) Mean value of the dynamic time constant 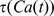 for each value of 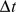 in panel B. The doublet protocol has little effect on 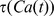. Curves correspond to those in panel B.

**Figure 4:**
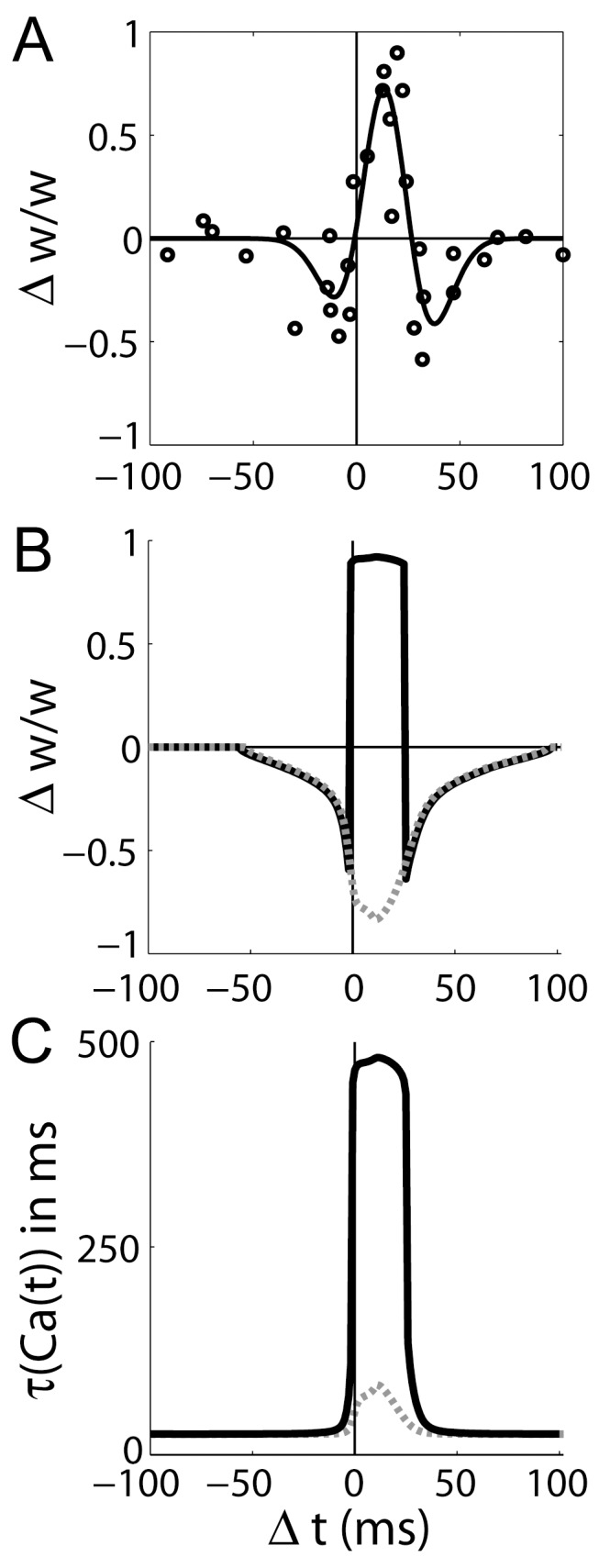
STDP at CA3-CA1 synapses with a single pre-synaptic spike and two post-synaptic spikes (the triplet protocol). (A) Data reproduced from [19], fit with a difference of Gaussians (least squares fit). 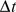 refers to time between the pre-synaptic spike and the second post-synaptic spike. (B) An inter-pairing frequency of 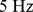 produced a ‘symmetric’ STDP curve, where a narrow LTP window is shifted in the causal (pre-before-post) direction, surrounded by two latency-dependent LTD windows (solid black). An inter-pairing frequency of 0.5 Hz produced a depression-only curve (dotted grey). The post-synaptic spikes were separated by 10 ms. (C) Mean value of the dynamic time constant 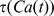 for each value of 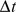 in panel B (corresponding curves).

The doublet protocol at 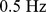 and 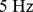 produced LTD-only curves, where the magnitude of LTD was greatest at low latency, decreasing with increasing latency in the causal (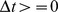) and anti-causal (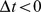) directions (Figure 3B). Very similar curves were produced for all frequencies less than or equal to 9 Hz. Quantitatively, these LTD-only curves are narrower than the Gaussian fit to the data shown in Figure 3A, but their qualitative resemblance is clear. The triplet protocol at 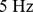 produced an LTP window for tight temporal coincidence (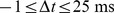), surrounded by LTD windows in which the magnitude of LTD decreased with increasing latency on either side of the LTP window (Figure 4B, solid black). The corresponding data are reproduced from [19] in Figure 4A. Note that a graded transition between maximal LTP and LTD can be produced by tuning the LTP threshold. The triplet protocol at 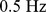 produced an LTD-only curve (Figure 4B, dotted grey). The mean value of the 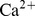-dependent 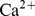 time constant 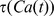 is plotted in Figures 3C and 4C for each condition shown in panel B in each figure.

**Figure 5:**
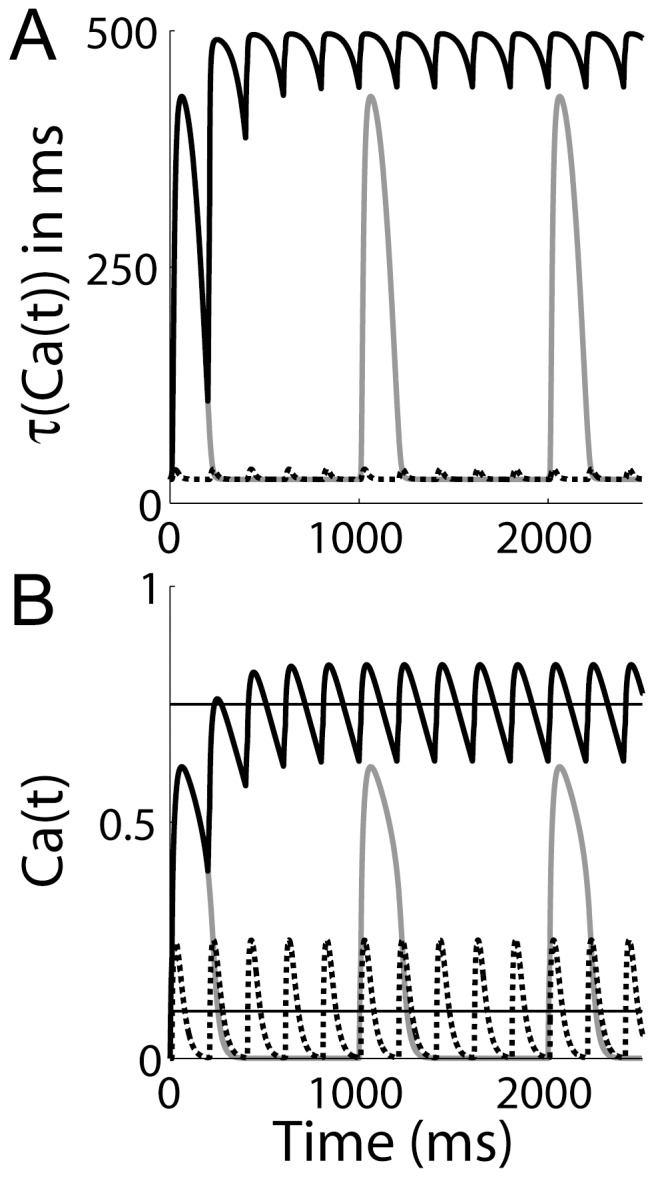
The 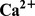-dependent 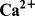 time constant 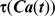 and corresponding values of the 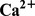-like variable 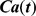 under the STDP protocols used by [19]. (A) The solid black curve shows 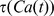 for pre-post-post spiking with 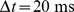 and an inter-pairing frequency of 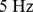, where 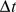 refers to the time between the pre-synaptic spike and the second post-synaptic spike. Post-synaptic spikes were separated by 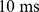. The grey curve shows 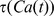 under the same spiking protocol, but with an inter-pairing frequency of 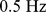. The dotted black curve shows 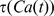 for pre-post spiking with 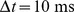 and an inter-pairing frequency of 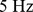, where 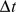 refers to the time between the pre-synaptic spike and the post-synaptic spike. (B) The 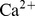-like variable 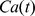 during the same simulations as panel A, with corresponding curves. The upper and lower horizontal lines show the LTP and LTD thresholds respectively.

Our simulations thus support the hypothesis that the post-synaptic burst-dependence and inter-pairing frequency dependence of LTP at CA3-CA1 synapses can explained by the saturation of the mechanisms of 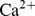 extrusion from the post-synaptic density. The mechanistic basis of these results is clear in Figure 5, where the dynamic time constant 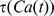 and the corresponding values of the 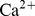-like variable 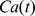 are shown for the above protocols. The dynamic time constant supports high levels of 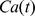 for the triplet protocol at 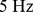, but under the other protocols, it decays to baseline between pairings (see figure caption for details).

### Simulations without the Dynamic 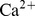 Time Constant

To determine whether the dynamic time constant 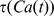 is required for the model to reproduce the data, we repeated our simulations without it, *i.e.* with 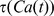 set to the baseline 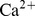 time constant 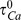. Because the parameter values given above were chosen for the intact model, it was necessary to choose parameter values without the dynamic time constant before running these simulations. To this end, we searched the space of values for three parameters: the baseline 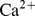 time constant 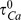, the time constant of decay of the BAP tail 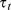, and the LTP threshold 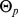. Both versions of the model (with and without the dynamic time constant) readily account for the LTD data under the doublet protocol, so we searched for parameter values that produce LTP under the triplet protocol at 5 Hz, but produce LTD under the triplet protocol at 0.5 Hz. We did not search the time constants of rise and decay of NMDAR activation because the LTP window shown by [19] was approximately 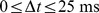, whereas the decay constant of NMDARs is 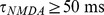, *i.e.* reasonable values of 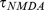 govern the width of causal (pre-before-post) LTD, but do not effect the occurrence of LTP. We did not search the scale factor 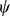 (Equation 5) because without the non-linearity of the dynamic time constant, this factor changes the scale of the simulations, not the phenomenology, *i.e.* any changes to 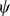 can be overcome by changes to 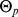. We did not search the proportion of the BAP given to the peak and the tail because these parameters change the scale of calcium transients for a given value of 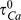, but not their decay time. As long as 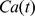 does not have the opportunity to buildup between pairings, LTP will not occur at 0.5 Hz. Note that the available data support our chosen values, justified in Methods section *Parameter values*. Finally, we ignored the learning rates 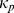 and 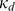 because they govern the magnitude of LTP and LTD respectively, not their occurrence.

**Figure 6:**
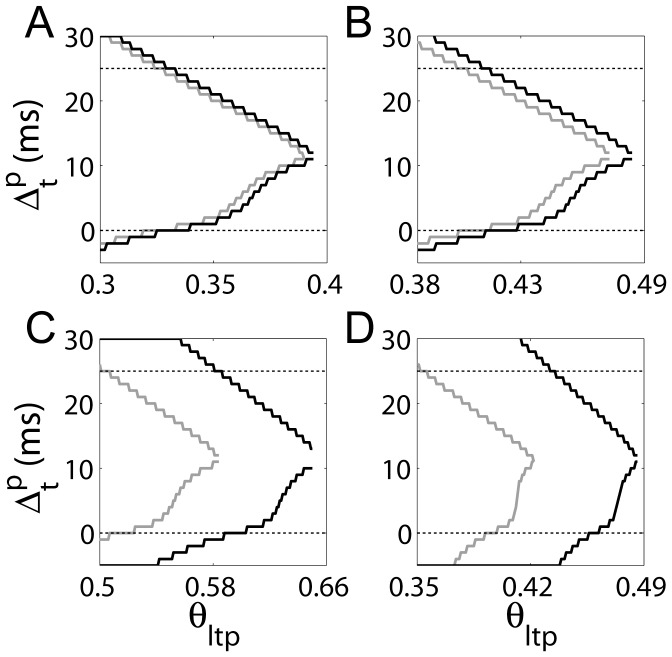
The LTP window 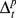 as a function of the LTP threshold 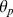 under the triplet protocol without the dynamic time constant (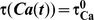). The time between the pre-synaptic spike and the second post-synaptic spike was 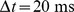. Post-synaptic spikes were separated by 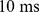. 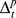 is defined by the onset (lower curves) and offset (upper curves) of LTP as a function of 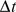, as shown in Figure 4. Black and grey curves show results for inter-pairing frequencies of 5 Hz and 0.5 Hz respectively. To be consistent with the data by [19], the black and grey curves should not overlap from 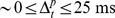. Without the dynamic time constant, the model requires biologically implausible parameter values to reproduce the data (panels C and D, see text). (A) The LTP window for parameter values justified in the Methods (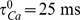, 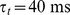). (B) The LTP window for 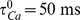 and 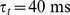. (C) An implausibly long baseline time constant of 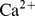 decay approximates the data (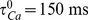, 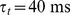). (D) An implausibly long time constant of decay of the BAP tail approximates the data, though 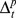 expands slightly in the anti-causal direction (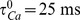, 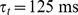).

The results of our parameter search for the model without the dynamic time constant are shown in Figure 6, where the LTP window (expressed as 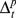) is shown over the LTP threshold 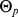 for different values of the baseline 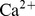 time constant 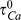 (panels A, B and C) and the BAP tail 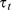 (panel D). The lower and upper curves show the lowest and highest values of 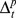 under the triplet protocol for a given set of parameters. The black and grey curves show results for 5 Hz and 0.5 Hz respectively, where 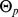 was incremented by 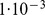 in the search. For a given set of parameters to be consistent with the data, there should be an LTP window between approximately 0 and 25 ms for 5 Hz, but not for 0.5 Hz, *i.e.* there should be black curves, but not grey curves in this region. For 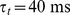 and 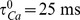 (justified in Methods section *Parameter values*), fine tuning of the LTP threshold permits a non-overlapping region for 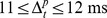 (Figure 6A). Doubling 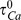 has little effect (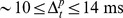, Figure 6B). The data can be approximately reproduced for 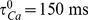 (Figure 6C), but this value is biologically implausible (see Methods section 0). Alternatively, with the plausible baseline 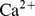 time constant 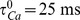, the data can be approximated with 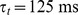 (Figure 6D), but we are unaware of any data to support a BAP tail longer than 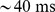. Thus, fine-tuning the model’s parameters allows the reproduction of the data without the dynamic time constant, but not under realistic parameter values. Further to this, we considered the robustness of the model by including noise in simulations of the triplet protocol at 0.5 Hz. Without the dynamic time constant, the model was given 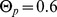 and the implausible baseline 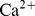 time constant 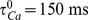 (see Figure 6C). We added normally distributed noise with mean 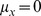 and standard deviation 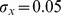 to each parameter 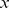 in each version of the model (with and without the dynamic time constant), and we ran 100 trials for each value of 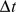 (the time between the pre-synaptic spike and the second post-synaptic spike) for 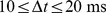 (increments of 1 ms). This range of 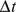 was used by [19] in their experiments with spike triplets at 0.5 Hz, producing LTD. The noise had little impact on the dynamic model, where only 6 out of 1100 trials produced LTP. Without the dynamic time constant, these simulations produced LTP on 256 trials out of 1100.

### Simulations of Further Spike-timing Protocols at CA1 Synapses

**Figure 7:**
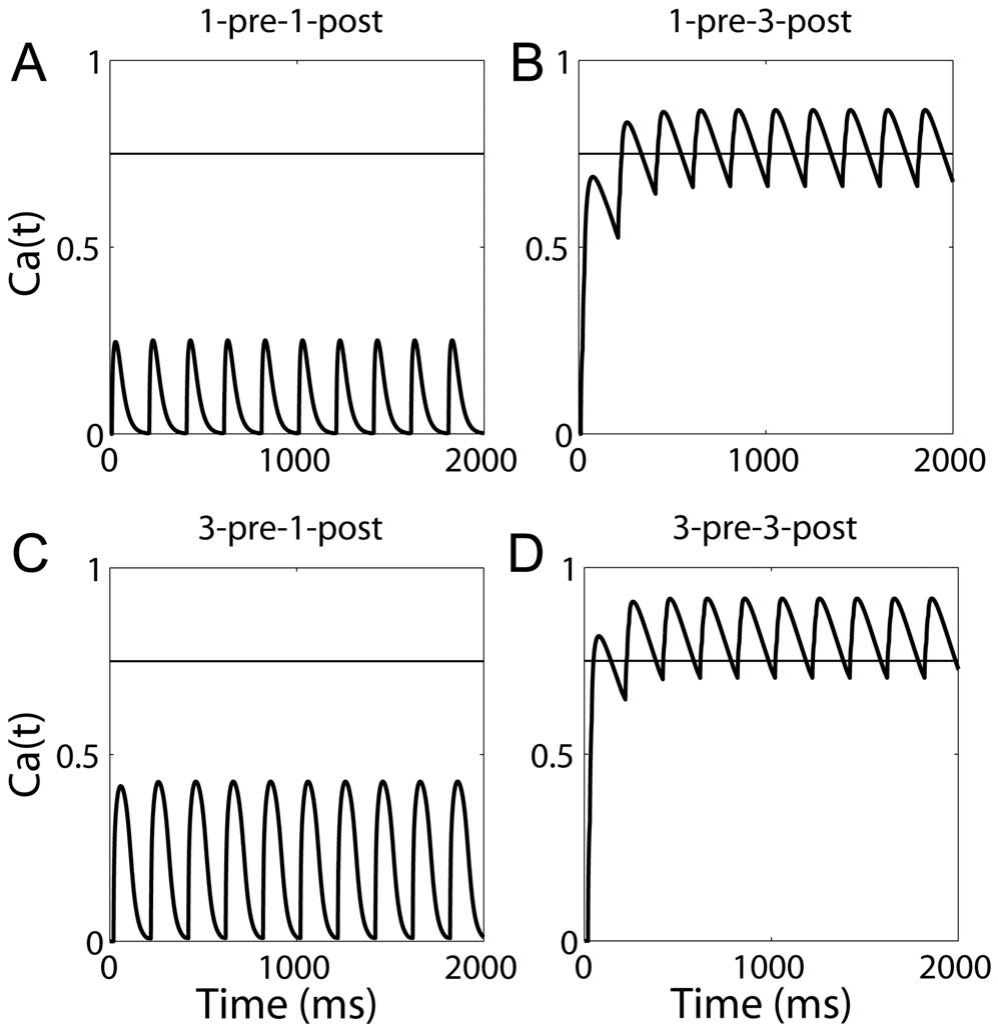
The 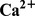-like variable 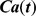 in simulations of the experiments by [36] Results are shown for simulations with 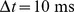, 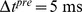 and 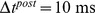, where 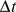 refers to the time between the last pre-synaptic spike and the first post-synaptic spike, 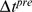 refers to the time between pre-synaptic spikes and 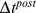 refers to the time between post-synaptic spikes. The LTP threshold (horizontal line) is only exceeded under conditions with post-synaptic bursting (1-pre-3-post and 3-pre-3-post), while pre-synaptic bursting has little effect on 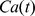 (3-pre-1-post). The inter-pairing frequency was 5 Hz.

The study by [19] is not the only one to demonstrate the requirement of post-synaptic bursting for LTP at CA1 synapses under spike-timing protocols with an inter-pairing frequency in the theta range. Earlier work by [36] also showed this requirement, where four protocols were followed: one pre-synaptic spike followed by one post-synaptic spike (1-pre-1-post), three pre-synaptic spikes followed by one post-synaptic spike (3-pre-1-post), one pre-synaptic spike followed by three post-synaptic spikes (1-pre-3-post), and three pre-synaptic spikes followed by three post-synaptic spikes (3-pre-3-post). For the 3-pre-1-post and 3-pre-3-post protocols, the time between pre-synaptic spikes was 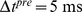. For the 1-pre-3-post and 3-pre-3-post protocols, post-synaptic spikes were elicited by current pulses of 20 ms, during which time the post-synaptic neuron emitted three spikes. In all cases, pre-synaptic activity preceded post-synaptic activity by 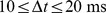. Ten pairings were provided with an inter-pairing frequency of 5 Hz, repeated four times at 10 s intervals. We simulated all four protocols for each integer value of 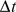. We set the time between post-synaptic spikes to 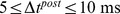, running simulations for each integer value within this range. For all 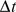 and 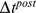, the model reproduced the finding by [36] that post-synaptic bursting is required for LTP. Figure 7 shows the corresponding values of 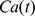 during ten pairings for 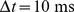 and 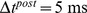. Note that [36] did not show LTD under these protocols, *i.e.* they showed no synaptic change, whereas the 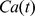 transients in Figure 7 for the 1-pre-1-post and 3-pre-1-post simulations exceed the LTD threshold used in our simulations of the experiments by [19]. Raising the LTD threshold from 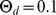 to approximately 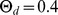 would reproduce this aspect of the data of [36]. Such an approach seems reasonable when comparing data from different studies, especially given the much higher 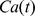 transients with post-synaptic bursting.

**Figure 8:**
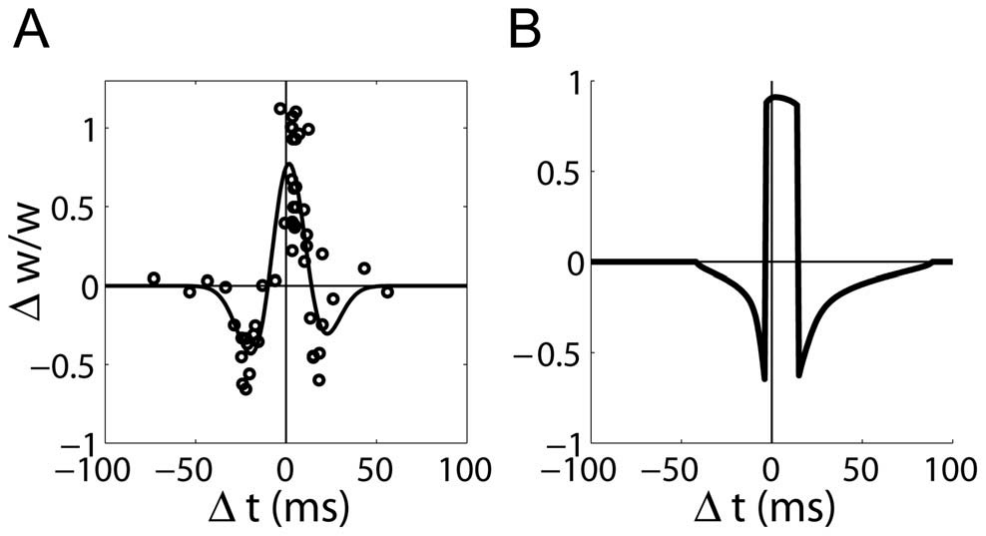
Temporally symmetric, bidirectional STDP under the double protocol at 5 Hz in a cesium-based intracellular solution (see text). (A) Data reproduced from [15], fit with a difference of Gaussians (least squares fit). (B) The effect of the cesium-based solution was simulated in the model by increasing the time constant of decay of the BAP peak by a factor of 5 (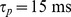)).

Where the study by [19] found that LTP at CA3-CA1 synapses required post-synaptic bursting and an inter-pairing frequency of 5 Hz, earlier authors found LTP at these synapses by pairing a single pre-synaptic spike with a single post-synaptic spike [15], *i.e.* the doublet protocol, also at 5 Hz. A possible explanation for this discrepancy was given by [19], who pointed to the use by [15] of an intracellular solution containing cesium instead of the normally-occurring potassium. Cesium blocks potassium channels, which can broaden action potentials and enhance BAPs in the apical dendrite, where CA3 neurons synapse onto CA1 neurons [57]. [19] therefore made further recordings under the doublet protocol (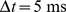) with a cesium-based intracellular solution, finding that action potentials were broadened by a factor of 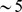. This manipulation was sufficient to rescue LTP. We simulated the effect of their cesium solution by broadening the decay constant of the BAP peak by a factor of 5 (from 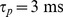 to 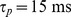) and re-running our simulations of the doublet protocol for the full range of latencies 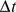. This small change to the model’s parameters was sufficient to qualitatively reproduce the data by [15] (Figure 8).

### Predictions for Experimental Enquiry

**Figure 9:**
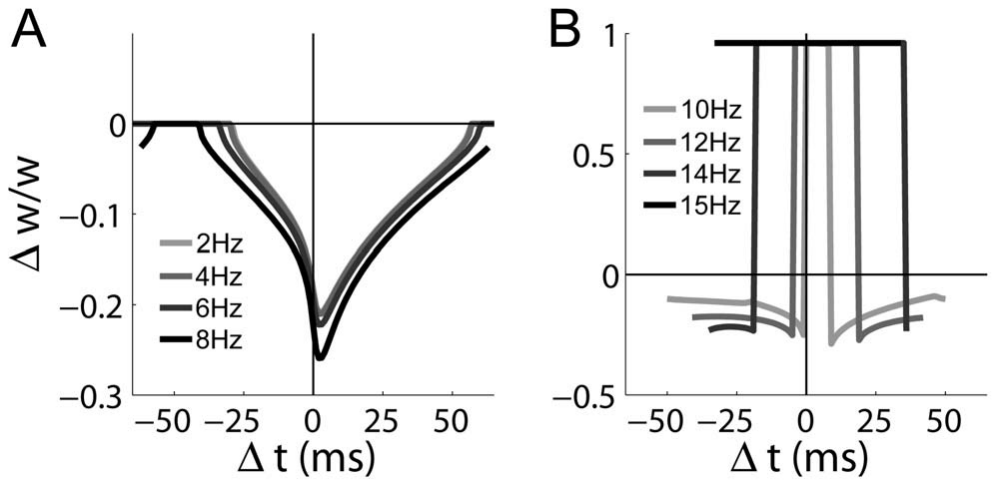
Predictions for STDP curves at CA3-CA1 synapses under the doublet protocol for a gradient of inter-pairing frequencies in the theta band (and higher). (A) 75 pairings at 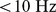 produced latency-dependent, LTD-only curves. (B) Temporally symmetric, bidirectional STDP was supported by frequencies from 10 Hz to 14 Hz (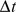 as defined in Figure 3). At 15 Hz, LTP is saturated for all latencies (horizontal black). In each panel, darker shades correspond to higher frequencies (see panel legends).

Having shown that the model accounts for the inter-pairing frequency dependence and post-synaptic burst dependence of LTP at CA3-CA1 synapses under the STDP protocols of [19], we ran further simulations to investigate more subtle variations of these two independent variables. First, we ran simulations of the doublet protocol at inter-pairing frequencies above 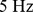. For frequencies 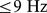, the model produced LTD-only curves (Figure 9A), similar to those shown in Figure 3B. A narrow, causal LTP window emerged at 10 Hz, expanding in both directions as frequency was increased up to 14 Hz, shown in Figure 9B. Note that the decreasing range of 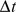 with increasing frequency in the figure reflects the shortening of the period. At 15 Hz, LTP was saturated for all 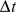 (Figure 9B). The model thus predicts that temporally symmetric, bidirectional STDP can be produced by the doublet protocol at higher frequencies within the theta band, including LTP under low-latency post-pre pairings, and that the spike timing-dependence of LTP is dominated by spike rate at frequencies higher than theta.

**Figure 10:**
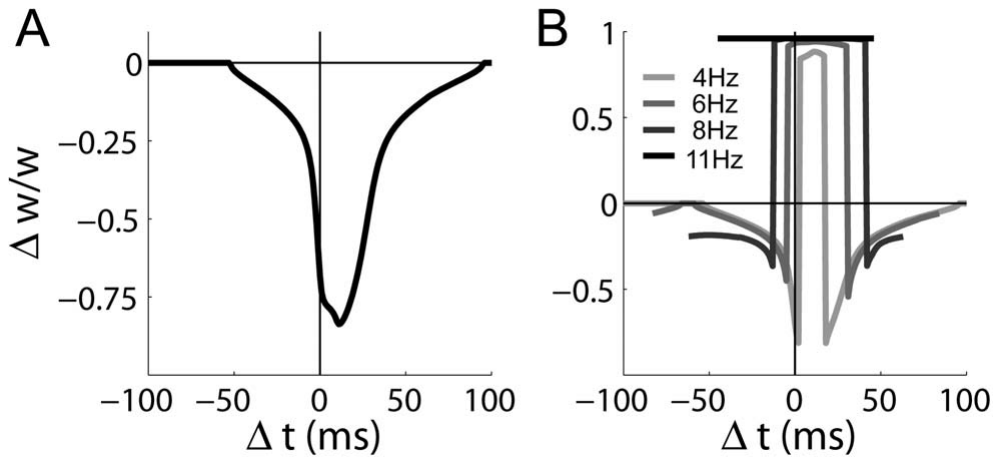
Predictions for STDP curves at CA3-CA1 synapses under the triplet protocol for a gradient of inter-pairing frequencies in the theta band (and lower). (A) 75 pairings at 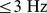 produced latency-dependent LTD-only curves. (B) Temporally symmetric, bidirectional STDP was supported by frequencies from 4 Hz to 10 Hz (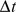 as defined in Figure 4). At 11 Hz, LTP is saturated for all latencies (horizontal black). Darker curves correspond to higher frequencies (see panel legend).

Next, we ran simulations of the triplet protocol with a range of inter-pairing frequencies. For frequencies 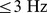, the model produced LTD-only curves (Figure 10A), identical to the dotted grey curve in Figure 4B. At 4 Hz, a narrow, causal LTP window emerged. Similar to the higher-frequency doublet simulations, this LTP window expanded in both directions as frequency was increased up to 10 Hz (Figure 10B). At 11 Hz, LTP was saturated for all 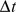 (Figure 10B). The model thus predicts that under the triplet protocol, the LTP window of temporally symmetric, bidirectional STDP expands in both directions at higher frequencies within the theta band, including LTP under post-post-pre pairings, and that the spike timing-dependence of LTP is dominated by spike rate at sufficiently high frequency.

**Figure 11:**
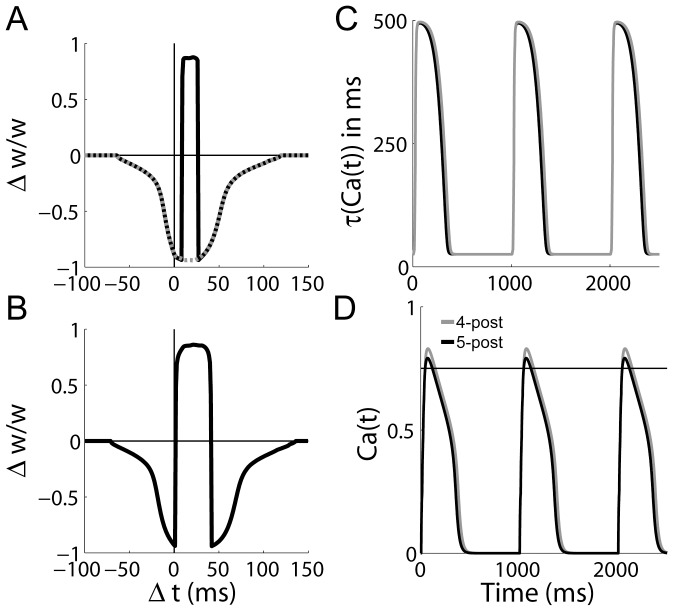
Predictions for STDP curves at CA3-CA1 synapses when a single pre-synaptic spike is paired with more than two post-synaptic spikes at inter-pairing frequencies lower than the theta band. (A) 75 pairings under the quadruplet protocol (3 post-synaptic spikes) at frequencies of 2 Hz (dotted grey) and 3 Hz (solid black). (B) 75 pairings under the quintuplet protocol (4 post-synaptic spikes) at 1 Hz and 2 Hz. The curves are indistinguishable. (C) The dynamic 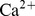 time constant 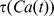 under the quintuplet and sextuplet (5 post-synaptic spikes) protocols at 1 Hz. Post-synaptic spiking followed the pre-synaptic spike by 5 ms. (D) 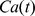 transients, where curves correspond to 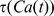 in panel C. The horizontal line shows the LTP threshold. In panels A and B, 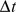 is defined as the time between the pre-synaptic spike and the last post-synaptic spike. In panels C and D, black and grey curves correspond to the quintuplet and sextuplet protocols respectively. Post-synaptic spikes were separated by 10 ms under all protocols.

Having made predictions for a gradient of frequencies under the doublet and triplet protocols, we investigated the post-synaptic burst-dependence of LTP under low-frequency protocols with more than 2 post-synaptic spikes. We ran simulations at 2 Hz and 3 Hz with a quadruplet protocol, where one pre-synaptic spike was paired with three post-synaptic spikes. As with the doublet and triplet protocols, 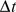 was defined as the temporal difference between the pre-synaptic spike and the last post-synaptic spike (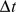 is also defined this way for quintuplet and sextuplet pairings below). Under the quadruplet protocol, an LTP window emerged at 3 Hz, shifted in the causal direction (LTP onset at 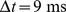, Figure 11A). At 2 Hz, a graded, LTD-only curve was produced by this protocol. Similarly, we ran simulations with a quintuplet protocol, where one pre-synaptic spike was paired with four post-synaptic spikes at frequencies of 1 Hz and 2 Hz. Temporally symmetric, bidirectional STDP was produced under these low inter-pairing frequencies (identical curves in Figure 11B). These predictions are important to the interpretation of the data by [19]. As shown in Figures 11C and D, there is a difference between the mechanism underlying LTP under the triplet protocol and under the quintuplet and sextuplet protocols (5 post-synaptic spikes). Under the triplet protocol, an inter-pairing frequency in the theta range prevents 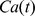 from decaying to baseline between pairings, so 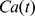 builds up over several pairings and eventually exceeds the LTP threshold (Figure 5). Under the quintuplet and sextuplet protocols, 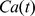 exceeds the LTP threshold during each post-synaptic burst, but decays to baseline between pairings. This mechanistic difference explains why the STDP curves under the quintuplet protocol were identical at 1 Hz and 2 Hz, *i.e.* the inter-pairing frequency made no difference (Figure 11B). The model thus predicts that with bursts of more than two or three post-synaptic spikes, LTP does not require theta-frequency pairings.

### Hebbian Associative Learning in the Network Model

**Figure 12:**
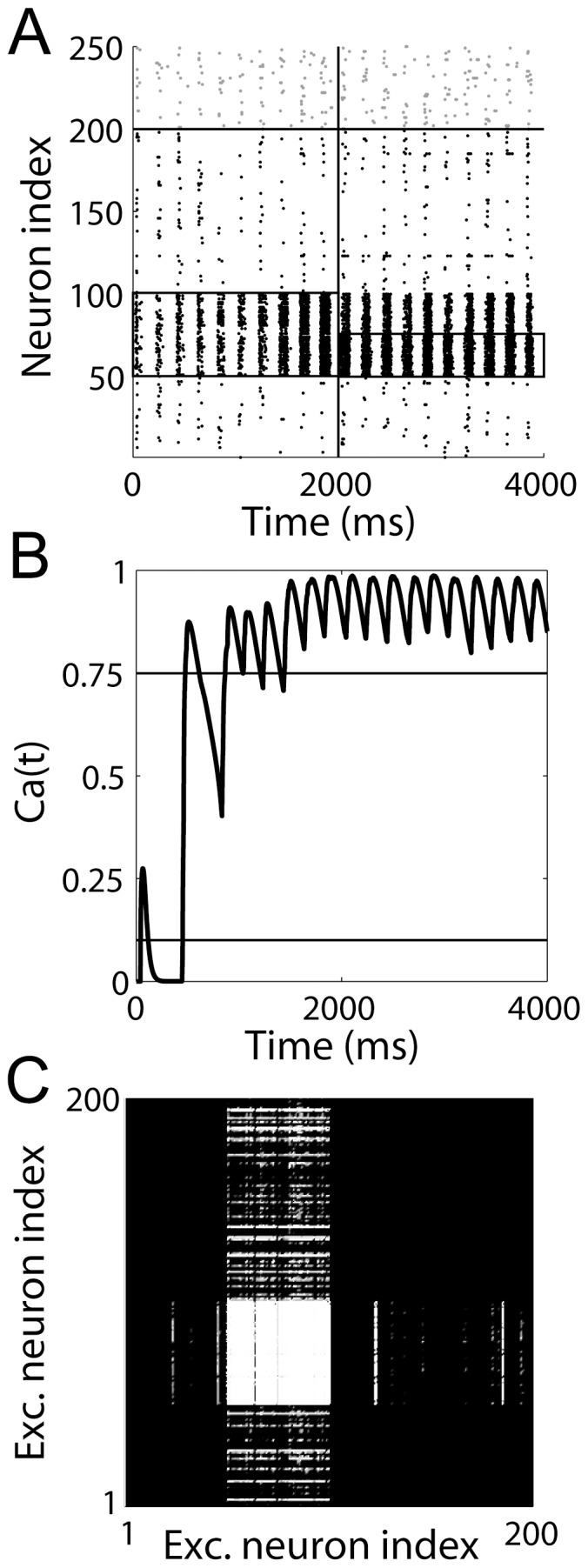
The 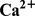 plasticity model supports Hebbian associative learning in a biophysical network model with realistic synaptic dynamics. (A) Raster plot showing network spiking during learning (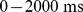) and auto-associative recall (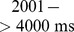). The vertical line distinguishes these stages of the trial. Pyramidal neurons and inhibitory interneurons are indexed from 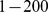 and 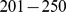 respectively. The upper horizontal line distinguishes these cell types. All neurons received noisy theta-band current injection (see text). Neurons 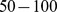 received Poisson spike trains at 500 Hz during the learning stage of the trial. Neurons 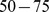 received this input during the recall stage. The population receiving the input spike trains is outlined with a thin black line. (B) The trajectory of the 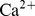-like variable 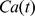 over the full trial at a randomly chosen synapse between two neurons in the selective population. Upper and lower horizontal lines show the LTP and LTD thresholds. (C) Synaptic conductance strength in the fully-connected network after learning, where lighter shades correspond to stronger synaptic connectivity.

Having shown that our plasticity model qualitatively reproduces STDP data from CA3-CA1 synapses [15], [19], [36] and makes predictions for experimental enquiry, we embedded the model in a network of integrate-and-fire neurons with realistic synaptic dynamics to determine whether it supports Hebbian associative learning under biophysically plausible conditions. Our chosen task was auto-associative recall, a classic task for hippocampal models [39], [40], [56], in which a spatial pattern of neural activity is associated with itself by the modification of excitatory recurrent synapses (see Discussion section *Synaptic plasticity, learning and memory*). All neurons in the network received noisy, theta-rhythmic current injection (see Methods section *Background activity*), while the neurons in a selective population (neurons 50–100 in Figure 12A) each received independent, homogeneous Poisson spike trains at 500 Hz, mediated by AMPAR conductance synapses. The input to the selective population was provided for 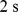 while the plasticity model was implemented at all recurrent synapses. We then provided the 500 Hz spike trains to half the selective population (neurons 75–100 in Figure 12A) for 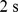 without the plasticity model. As shown in Figure 12, the model supported associative learning, *i.e.* the network recalled the full spatial activity pattern from partial input. The value of 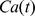 at a randomly-chosen synapse between two neurons in the selective population is shown in Figure 12B. The final strength of all synapses in the network is shown in Figure 12C.

## Discussion

Under STDP protocols, LTP at CA3-CA1 synapses depends on post-synaptic bursting and an inter-pairing frequency in the range of the hippocampal theta rhythm [19]. We hypothesize that these dependencies reflect the saturation of the mechanisms of 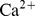 extrusion from the post-synaptic spine. We have tested this hypothesis with a minimal model of 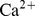-dependent plasticity, where the saturation of the mechanisms of 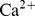 extrusion is simulated by the 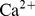-dependence of the time constant of 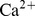 decay (Figure 1). Our model qualitatively reproduces the STDP data of [19], where latency-dependent LTD is elicited by pre- and post-synaptic spike doublets at frequencies of 5 Hz or less (Figure 3), and by spike triplets (one pre-synaptic spike and two post-synaptic spikes) at frequencies of 0.5 Hz or less (Figure 4). At a frequency of 5 Hz, triplet pairings produce temporally symmetric, bidirectional STDP (Figure 4). Without the 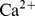-dependent 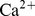 time constant, the minimal model cannot reproduce these data under biologically plausible parameter values (Figure 6). The model further accounts for the dependence of LTP on post-synaptic bursting at CA1 synapses under other spike timing protocols, including those with multiple pre-synaptic spikes [36] (Figure 7); and accounts for temporally symmetric, bidirectional STDP under spike doublets with intracellular solutions that broaden BAPs at CA3-CA1 synapses [15], [19] (Figure 8).

The model makes testable predictions about the burst-dependence and frequency-dependence of STDP at CA3-CA1 synapses. For example, our simulations predict that temporally symmetric, bidirectional STDP will emerge under the doublet protocol at higher frequencies within the theta band (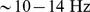), with the LTP window expanding in the causal and anti-causal directions with increasing frequency (Figure 9). The model predicts that this expansion will also occur under the triplet protocol at lower theta frequencies (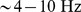, Figure 10) and that pairing pre-synaptic spikes with more than two or three post-synaptic spikes will elicit LTP at any sub-theta frequency (Figure 11). Our 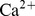 plasticity model is simple enough to be implemented at the network level and we have demonstrated associative learning and recall under theta oscillations in a biophysical network model with realistic synaptic dynamics (Figure 12).

Our model is based on the 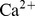 control hypothesis [23], [30], where high levels of post-synaptic 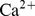 lead to LTP and moderate levels lead to LTD. Consistent with the NMDAR-dependence of plasticity at CA3-CA1 synapses, NMDARs provide the relevant source of 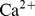. We refer to our model as ‘minimal’ because the only biophysical mechanisms included in the model are those required to reproduce the data being addressed: a marker for pre-synaptic spikes, a marker for post-synaptic spikes, and a variable to quantify the coincidence of these markers, the time constant of which is monotonically dependent on the magnitude of the variable. The respective biophysical correlates of these computational requirements are NMDARs, long-tailed BAPs, 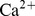, and the 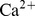-dependent 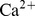 time constant 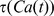. This parsimonious approach not only limits the assumptions of the model, but affords a simple implementation at the network level. Indeed, the chosen network model is endowed with the minimum biophysical detail to account for a range of data from perceptual and cognitive tasks (*e.g.* [45], [51], [52], [58]). The only biophysical mechanism that we added to this generic network is the BAP, driving 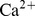 dynamics in conjunction with NMDARs.

### STDP and the Need for Physiologically-motivated Models

Since the discovery of graded, temporally asymmetric STDP in cultured hippocampal cells [14], numerous computational models have sought to characterize synaptic plasticity according to these data. In much of this work, ‘STDP rules’ have been defined by curves fit to the data, which are assumed to capture the phenomenology of synaptic change with ongoing pre- and post-synaptic spikes [59]–[64]. This assumption is perfectly reasonable, indeed unavoidable, for the spike trains under which the data were recorded. Given the implicitly Hebbian form of temporally asymmetric STDP, this approach has an intuitive appeal, *i.e.* pre-post spike doublets imply that a pre-synaptic cell ‘repeatedly and persistently takes part in firing’ [1] a post-synaptic cell and should therefore lead to LTP, whereas post-pre doublets imply a lack of causality and therefore LTD. Further appeal stems from competitive interactions between the LTP and LTD portions of asymmetric STDP curves, known to support synaptic scaling with uncorrelated [65] and correlated [64] pre- and post-synaptic spiking.

The application of asymmetric STDP rules to spike trains different from those that produced the underlying data has not been straightforward. In neural simulations, the sampling of STDP curves with each pre- and post-synaptic spike has not accurately predicted the outcome of various triplet, quadruplet, and quintuplet protocols (see [22]). Augmenting STDP rules with so-called spike interaction rules has been productive in this regard, limiting the contribution of individual spikes to plasticity [17], [62], [66]. Biological correlates have been proposed for the time-dependent suppression of interactions between spikes [67], but ultimately, this approach requires the continual addition of rules to accommodate data recorded under different spiking conditions. See [22] for a thorough treatment of the limitations of this phenomenological approach. Note that temporally symmetric, bidirectional STDP [15] was discovered near-concurrently to its asymmetric homologue, but its phenomenology has received little attention.

#### 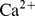-based plasticity models

An alternative to the phenomenological approach is to model synaptic physiology, providing a mechanistic explanation for plasticity data recorded under any protocol. STDP data have motivated several such models, including ours. As described above, all such models are based on the 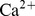 control hypothesis, where high and moderate levels of 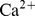 lead to LTP and LTD via kinase- and phosphatase-activated pathways respectively. Our minimal model simulates 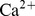 transients by the product of NMDAR activation and the BAP, where the 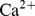-dependent 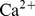 time constant 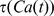 captures the saturation of the mechanisms of post-synaptic 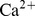 extrusion. Earlier 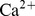 models differ along a number of dimensions, including their underlying assumptions, their levels of biophysical detail, and the data they address. All of these models assume that NMDARs provide 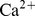 for plasticity, but some assume that voltage-gated 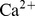 channels (VGCCs) are also required (e.g. [24]–[26], [29]), an assumption supported by experimental data under some conditions [68], [69]). Assumptions differ about the relevant aspect of supra-threshold 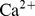, such as integrated 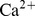 [23], [27], [29], [30], [33] and the peak [24], [28] and timecourse [25], [26] of 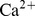 transients.


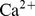
-based models have been used to address temporally symmetric and asymmetric STDP data. Any model based only on post-synaptic 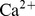 levels will demonstrate anti-causal LTD [23], [26] and will therefore account for symmetric STDP. Additional mechanisms are required to explain asymmetric STDP, for example, the inclusion of separate sources of 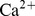 for LTP and LTD processes [24], [27] or competitive interactions between these processes and an LTD veto process for appropriate 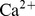 transients [26]. The former is supported by data from neocortical slices [70] and the latter is consistent with competition between kinases and phosphatases (*e.g.* [71]). Since BAPs are known to attenuate with dendtric distance from the soma, several studies have considered plasticity outcomes as a function of dendritic location [29], [30], [33], accounting for experimental data showing a switch from LTP to LTD at longer distances [72].

Like our model, some existing 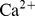-based models have simulated LTP and LTD directly from supra-threshold levels of 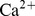 [23], [24], [28], [33], but others have included an intermediate processes for each of LTP and LTD [26], [27], [29], [30]. These processes correspond to intracellular signalling cascades that mediate plasticity outcomes from 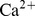 levels, which may be loosely equated with kinase- and phosphatase-activated pathways respectively. Two recent studies augmented this approach with a mechanism for synaptic bistability, addressing STDP data. In the model by [29], post-synaptic 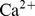 is the sum of independent pre- and post-synaptic 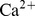 components, assumed to be mediated by NMDARs and VGCCs respectively. Their 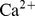 variable is thus incremented with every pre- or post-synaptic spike, increasing or decreasing the efficacy of bistable synapses when it exceeds the respective thresholds for LTP and LTD. While their 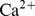 variable has a time constant of a few milliseconds (like 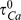 here), their synaptic changes occur on a timescale on the order of minutes, driving all-or-none transitions to a down-state or an up-state, to which the efficacy variable relaxes in the absence of synaptic activity. Their model reproduces a broad range of data by varying the magnitude of the independent pre- and post-synaptic 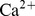 components, the LTP and LTD thresholds, and the learning rates for LTP and LTD. In the model by [30], supra-threshold 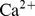 transients change the probability of switching from the down-state to the up-state or *vice versa*, where these changes tend to occur following spiking activity.

As a model of CA1 plasticity, our model addresses temporally symmetric STDP, where the 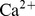-dependence of 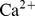 decay [35] accounts for the frequency and burst-dependence of LTP [19]. The coincidence of NMDAR activation and the BAP provides the only source 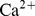 in our model. Above the thresholds for LTD and LTP, 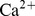 is integrated, decreasing and increasing simulated synaptic strength respectively. Note that we do not claim that 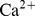 dependent 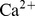 decay is the only mechanism that could possibly account for the frequency and burst dependencies of LTP, but we have shown that under our minimalist assumptions, the model requires implausible parameter values to account for these dependencies without the proposed mechanism. A concise comparison of the above models and ours is provided by Table 1.

It is worth noting that several authors have developed plasticity models that occupy the middle ground between phenomenological and physiological models. Under this approach, abstract variables are responsive to pre- and post-synaptic activity and their values serve as parameters to learning rules that determine changes to synaptic weights. These models have successfully generalized across timing- and rate-based plasticity protocols [73], [74].

#### The timecourse of supra-threshold calcium transients for LTP and LTD

It has been suggested that high levels of 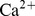 leading to LTP can be brief, but that moderate levels leading to LTD must be prolonged (*e.g.* [26], [75]). The technical challenges of controlling and measuring 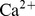 flux at individual synapses are considerable and direct evidence for or against this suggestion is consequently sparse. Perhaps most convincingly, [76] used 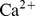 ‘uncaging’ to control post-synaptic 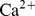 elevation over tens of seconds, where high elevations over 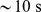 produced LTP and moderate elevations over 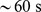 produced LTD. These authors noted that the magnitude and duration of 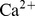 elevation comprise a two-dimensional space that is largely unexplored. For instance, it is unclear whether a high-magnitude elevation over a long timescale (*e.g.* 60 s) would lead to LTP. To the best of our knowledge, this possibility has not been addressed. The flip side of this issue is whether LTD requires 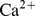 to remain above the LTD threshold for an unbroken period of time, as suggested by [26], or simply requires more repetitions of a given protocol than LTP, *i.e* a higher number of discrete supra-threshold 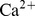 transients. If the latter, then the slow onset of LTD under several protocols (*e.g.* [9], [19], [77]) can be explained by a low LTD learning rate (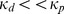 in our model). Notably, at least two studies have shown 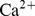 transients that reach higher amplitudes and decay *more slowly* with LTP-inducing stimuli than with LTD-inducing stimuli [78], [79]. Our model is very robust to parametric variation and our results can be obtained qualitatively for parameters under which 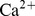 exceeds the LTP threshold either in discrete jumps or for an unbroken interval. For 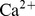 to remain above the LTD threshold for an unbroken interval, an additional mechanism is required, *e.g.* VGCCs. This characterization is apparent in Figure 5, where the 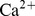-dependence of the 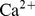 time constant prevents high levels of 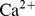 from undergoing significant decay, but moderate levels of 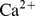 drop to baseline between pairings. Methods for controlling and measuring post-synaptic 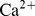 are continually improving (see [47]), so we expect these considerations will be addressed in due course.

### Addressing Data from Different Plasticity Protocols

We have stated that 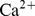-based models capture the underlying physiology of plasticity and are therefore able to address data recorded under different protocols. Several studies have taken this approach (*e.g.* [25], [29]), though it is unclear that a given model should account for data recorded from different tissue preparations and brain regions, *i.e.* ‘caution is warranted when generalizing from one synapse to another’ [32]. We have also stated that our model contains the minimal biophysical detail to explain the post-synaptic burst-dependence and inter-pairing frequency-dependence of LTP at CA3-CA1 synapses under STDP protocols [19]. Notably, no additional mechanisms or parameter changes were required for the model to reproduce the major findings of studies in which axonal tracts onto CA1 neurons were subject to tetanic stimulation. These studies showed that a brief period of high-rate stimulation (*e.g.* 100 Hz stimulation for 1 s) produces LTP and a long period of low-rate stimulation (*e.g.* 1 Hz for 1000 s) produces LTD [9], [19]. It is not surprising that we were able to reproduce these findings with an integrate-and-fire neuron. If pre-synaptic stimulation is strong enough to drive the neuron, then low-rate stimulation will lead to LTD in our model (Figures 3B, 9A and 10A). Under the same parameters, high-rate stimulation effectively produces multiple low-latency, pre- and post-synaptic spikes, leading to LTP (not shown).

**Figure 13:**
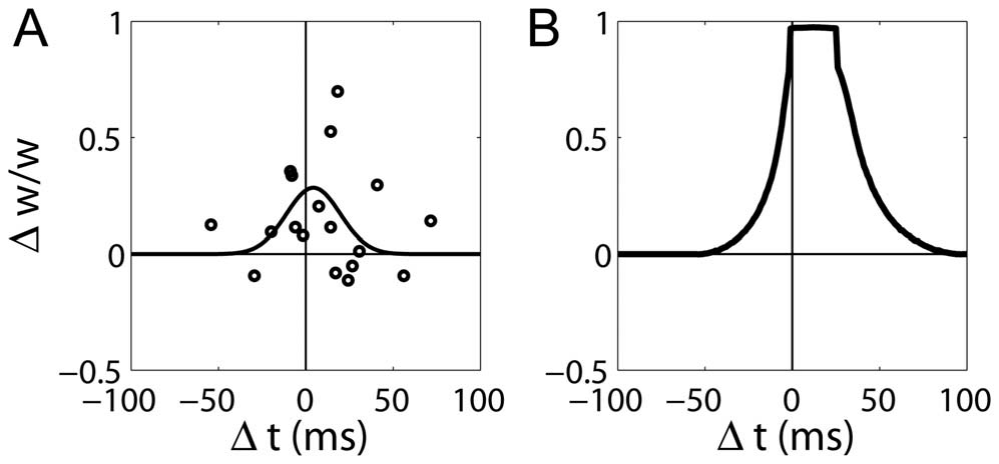
LTP-only curves for a small number of pairings under the triplet protocol at 5 Hz. (A) Data reproduced from [19], fit with a Gaussian function (lease squares fit). (B) LTP ‘fatigue’ produces an LTP-only curve for 25 pairings under the triplet protocol. Fatigue of the processes initiating LTP was simulated with an LTP-dependent LTP threshold 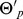, where 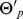 was given an initial value equal to the LTD threshold 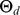, to which it decayed with a time constant of 1 s. For all positive values of 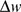, 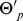 was incremented by 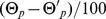. All other parameters are identical to the simulations above. 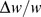 refers to the change in synaptic strength relative to initial strength (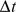 defined as in Figure 4).

An aspect of the data by [19] that we did not address above is the dependence of STDP on the number of triplet pairings. Specifically, these authors showed that with 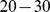 triplet pairings (instead of 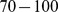), their data were fit with a latency-dependent LTP-only curve. These data can be explained by several mechanisms. For example, [29] allowed each of their independent pre- and post-synaptic 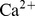 transients to exceed the LTP threshold. Another possibility is short term fatigue of the mechanisms initiating LTP, readily simulated in our model by increasing the LTP threshold with ongoing LTP. To demonstrate this mechanism, we ran 25 pairings under the triplet protocol with an LTP-dependent LTP threshold. The resulting LTP-only curve is shown in Figure 13 (see the caption for details).

### Synaptic Plasticity, Learning and Memory

The use of biophysical plasticity models in network simulations is an important direction in neuroscience, allowing the investigation of the network- and systems-level consequences of plasticity processes. Network- and systems-level modelling with synaptic resolution has a fruitful recent history. For example, our understanding of the respective roles of AMPARs and NMDARs in cortical processing has been greatly advanced by simulations of persistent mnemonic activity in working memory tasks [44], [51], [80], the integration of evidence in decision tasks [45], [81], and the role of oscillations in the transfer of information between cortical networks [58], [82]. Several recent papers have discussed the need for this level of biophysical resolution in network models with plastic synapses [22], [29], [30]. Our simulations of associative memory show that our plasticity model is tractable in this regard. We are aware of only one model to previously bridge this gap, doing so under different assumptions than ours and performing a different learning task [27].

Our 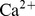 plasticity model is based on data from CA3-CA1 synapses [19], [36], [83], but memory formation in our network model is perhaps more easily equated with plasticity at CA3-CA3 synapses. Hippocampal region CA3 has long been hypothesized to support episodic memory by auto-association, owing to the dense lateral synaptic connectivity among CA3 neurons and to the well-established role of the hippocampus in episodic memory. For review of this hypothesis, see [84]. The use of our plasticity model in this task seems reasonable, as there do not appear to be any substantial physiological or plasticity differences between CA3-CA3 and CA3-CA1 synapses (Alan Fine, personal correspondence). It is worth noting that temporally symmetric STDP is strikingly consistent with the hypothesis that CA3 supports auto-associative recall. For example, in the hippocampal models by Lisman, Jensen and colleagues, theta/gamma oscillations synchronize coupled neurons and memories are encoded when tight temporal coincidence of pre- and post-synaptic spiking leads to LTP and loose coincidence leads to LTD [56], [85]. These conditions are captured by symmetric STDP data from hippocampal preparations [15], [19]. In this regard, it is perhaps surprising that the phenomenological implications of symmetric STDP have not received more attention.

### Conclusions and Future Work

Our plasticity model builds on earlier work that identified the minimal mechanisms for NMDAR-dependent synaptic plasticity: markers for pre- and post-synaptic activation and a variable (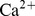) to capture their timing correlations [23]. We have augmented this well-established approach with a previously-unexplored computational mechanism, namely the 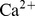-dependence of 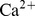 decay. This dynamic 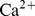 time constant captures the saturation of the mechanisms of 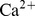 extrusion from the post-synaptic spine [34], [35]. Our simulations of STDP experiments demonstrate that this mechanism is sufficient to explain the burst- and frequency-dependence of LTP at CA3-CA1 synapses [19]. Our simulations of untried STDP protocols make testable predictions for the implications of the proposed mechanism and our simulations of associative learning demonstrate that our plasticity model is tractable at the network level.

A limitation of our minimalist approach is the requirement of the BAP for plasticity. As such, the present model cannot reproduce plasticity data obtained under protocols that hold post-synaptic neurons at fixed levels of depolarization for prolonged periods (see [86]). Furthermore, BAPs are neither necessary nor sufficient for plasticity under some conditions [21]. More generally, our 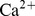-like variable is the product of the post-synaptic BAP and pre-synaptic activation, so our model is limited to homo-synaptic plasticity. These considerations point to the need for studies addressing the functional consequences of different plasticity phenomena. The suitability of symmetric STDP to auto-associative learning provides an example of such a functional consequence.

Our study points to several lines of enquiry for future work. Our implementation of the 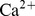-dependence of 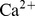 decay is purposefully simplified and a detailed implementation of the mechanisms of 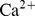 extrusion may reveal the limitations of our more abstract approach. If so, it may also reveal the computational properties of unexplored calcium dynamics and determine the conditions under which our present, efficient implementation is justifiable in network simulations.

Finally, our study is consistent with the hypothesis that synaptic plasticity is regulated by the modulation of 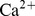 extrusion from dendritic spines. In support of this hypothesis, a recent study demonstrated that LTP is not expressed by pyramidal neurons in hippocampal region CA2 largely because rates of extrusion are significantly higher than in regions CA1 and CA3, which express LTP [48]. In these experiments, LTP was blocked in CA1 and induced in CA2 by interventions that speed up and ‘overwhelm’ 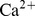 extrusion respectively. We hypothesize that LTP is *only* produced if the mechanisms of extrusion are overwhelmed. Biophysical mechanisms that may control the saturation of 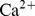 extrusion constitute an exciting direction for future research on synaptic plasticity and its functional outcomes.
